# Dietary Whole Wheat Flour Intake in Pakistan: A Cross-Sectional Insight into Long-Term Dementia Prevention

**DOI:** 10.3390/nu18071135

**Published:** 2026-04-01

**Authors:** Fatima Masood, Antonio Minò, Fouzia Sadiq, Riaz Mahmood, Alfonso Di Costanzo, Antonella Angiolillo

**Affiliations:** 1 Department of Medicine and Health Sciences “V. Tiberio”, Centre for Research and Training in Medicine of Aging, University of Molise, 86100 Campobasso, Italy; fatimamasoodofficial@gmail.com (F.M.); antonio.mino@unimol.it (A.M.); alfonso.dicostanzo@unimol.it (A.D.C.); 2 Directorate of Research, Shifa Tameer-e-Millat University, Islamabad 44000, Pakistan; fsadiq09@gmail.com; 3 Department of Neurology, Federal Government PolyClinic Hospital, Islamabad 44000, Pakistan; riazgem1@yahoo.com; 4 Molise Regional Health Service (ASREM), 86100 Campobasso, Italy

**Keywords:** whole wheat flour, dietary habits, dementia, cognitive health, prevention

## Abstract

**Background/Objectives**: Whole grains are increasingly recognized as protective components of dietary patterns linked to healthy aging and reduced risk of chronic disease. Nevertheless, relatively limited research has explored the relationship between whole wheat flour consumption and cognitive health in South Asian populations, where wheat-based foods represent a major dietary staple. This study investigated the association between habitual whole wheat flour intake and cognitive status in a Pakistani population. **Methods**: A two-phase cross-sectional study was conducted using interviewer-administered dietary questionnaires. In the first phase, dietary habits related to wheat product consumption were assessed in a population sample of 144 adults. In the second phase, dietary profiles were compared between two matched groups: cognitively healthy individuals (HLT, *n* = 30) and patients with clinically diagnosed dementia (PwD, *n* = 30). Categorical variables were analyzed using Pearson’s chi-square and Fisher’s exact tests. **Results**: Whole wheat flour was the predominant flour type used among respondents. Compared with PwD, HLT reported significantly higher consumption of whole wheat flour and greater adherence to dietary practices associated with whole grain intake. HLT also reported higher consumption of several foods commonly associated with brain-supportive dietary patterns. **Conclusions**: Although causal relationships cannot be inferred due to the cross-sectional design, the findings suggest that whole wheat flour consumption may be associated with broader dietary patterns linked to cognitive health. Given the central role of wheat-based foods in the Pakistan diet, promoting whole wheat flour as a staple food choice may represent a culturally feasible strategy within dietary approaches aimed at supporting healthy brain aging.

## 1. Introduction

Dementia represents a growing global public health challenge, with increasing prevalence in aging populations and substantial social and economic burden. According to the World Health Organization (WHO) and Alzheimer’s Disease International, more than 55 million people worldwide currently live with dementia, a number projected to rise to more than 150 million by 2050, largely driven by population aging in low- and middle-income countries. Globally, dementia affects approximately 5–8% of individuals aged 65 years and older, with prevalence increasing steeply with age [1].

In the WHO Southeast Asia Region, pooled data from population-based studies estimate an overall dementia prevalence of about 3% among individuals aged 60 years and older. Prevalence increases markedly with age, ranging from approximately 1 to 2% in those aged 60–69 to more than 10–12% among individuals aged 80 and above. Owing to rapid demographic changes, the total number of people living with dementia in Southeast Asia is projected to rise considerably in the coming decades, underscoring the region’s expanding contribution to the global burden of dementia [2,3].

Preventive strategies are urgently needed, particularly those based on modifiable lifestyle factors such as diet [4]. Among these, dietary patterns that emphasize whole grains have attracted considerable interest, as they may confer neuroprotective benefits via multiple mechanisms, including reduced inflammation, improved vascular health, and metabolic regulation [5,6].

Several epidemiological studies suggest that higher whole grain consumption is associated with a lower risk of cognitive decline and dementia. For example, in the Framingham Offspring Cohort, individuals with the highest intake of whole grains had a significantly lower risk of developing dementia and Alzheimer’s disease compared to those with the lowest intake [6]. In addition, in a biracial cohort drawn from the Chicago Health and Aging Project, greater whole grain consumption was linked to a slower rate of decline in global cognition, perceptual speed, and episodic memory, especially among African American participants [7].

Systematic reviews of observational and interventional studies further explore these associations. Though the overall evidence is still considered limited and of low-to-moderate strength, several studies point to potential cognitive benefits of whole grain intake [8]. Moreover, population-based reviews of dietary patterns (such as the Mediterranean and MIND diets), which often include whole grains as a core component, consistently report neuroprotective effects and reduced incidence of cognitive disorders [9,10].

At a biological level, whole grains are rich in dietary fiber, B vitamins, polyphenols, and other bioactive compounds which may contribute to neuroprotection. These nutrients have been implicated in mechanisms such as antioxidant activity, reduced oxidative stress, and improved endothelial function, all relevant to delaying or preventing neurodegenerative processes [11].

While growing evidence suggests that whole grain consumption may contribute to metabolic health and reduced inflammation, its potential relationship with cognitive health remains insufficiently explored, particularly in South Asian populations where wheat-based foods represent a major dietary staple. Most previous studies have focused on western dietary patterns or specific nutrients, whereas limited attention has been given to culturally specific staple foods such as whole wheat flour and their possible association with dementia risk. Therefore, understanding the relationship between habitual consumption of whole wheat products and cognitive health may provide relevant insights for population-specific dietary prevention strategies.

Based on these considerations, we hypothesized that long-term dietary habits related to whole wheat flour consumption may differ between cognitively healthy individuals (HLT) and patients with dementia (PwD), potentially reflecting dietary patterns associated with cognitive resilience.

Therefore, the aim of the present study was to investigate the association between habitual whole wheat flour consumption and dementia status in a Pakistani population, using a questionnaire-based assessment of long-term dietary habits. In the first phase, a population-level survey was conducted to assess general dietary patterns and the use of wheat-based products. In the second phase, a comparative analysis was performed between HLT and PwD to examine differences in dietary behaviors, awareness of brain-healthy foods, and factors associated with cognitive health.

## 2. Materials and Methods

### 2.1. Study Design

This study employed a two-phase, cross-sectional design to explore dietary whole wheat flour consumption patterns in Pakistan and their potential association with long-term dementia prevention. Data were collected using structured, interviewer-administered questionnaires focused on dietary habits and whole wheat flour intake.

#### 2.1.1. Phase I: Population-Level Dietary Survey

In the initial phase, 144 adult participants were recruited from various urban and semi-urban regions of Pakistan. Individuals aged ≥18 years and willing to provide informed consent were eligible for inclusion. The survey assessed general dietary patterns, frequency of whole wheat flour consumption, socioeconomic characteristics, and lifestyle factors. The demographic and clinical characteristics of the study group are presented in Table 1. Participants were interviewed in person, and all responses were recorded using a questionnaire developed for this study.

#### 2.1.2. Phase II: Case–Control Dietary Assessment

In the second phase, 60 participants were enrolled, comprising 30 PwD clinically diagnosed and 30 HLT controls matched for age and sex. All recruited participants underwent initial cognitive screening using the Mini-Cog test to assess cognitive status [12]. Those who screened positive were subsequently evaluated by a qualified neurologist at Shifa International Hospital (Islamabad) and underwent comprehensive clinical and neuropsychological assessment. Dementia diagnosis was established according to the National Institute on Aging/Alzheimer’s Association (NIA–AA) criteria [13]. The Mini-Mental State Examination (MMSE), used to assess dementia severity, indicated that all patients had mild-to-moderate dementia, with scores ranging from 24 to 16 [14].

HLT had no history of cognitive impairment and scored within the normal range on routine cognitive screening. All participants in this phase completed the same dietary questionnaire used in Phase I, with additional questions focusing on long-term whole wheat flour intake, concomitant dietary habits, and medical history. Caregivers (family members in almost all cases) assisted patients with the questionnaire to ensure accurate reporting. The demographic and clinical characteristics of PwD and HLT controls are presented in Table 2.

### 2.2. Ethical Considerations

The study was conducted in accordance with the Declaration of Helsinki and approved by the appropriate Institutional Review Board & Ethical Committee (IRB&EC) of Shifa Tameer-e-Millat University (IRB # 367–24). Written informed consent was obtained from all participants or, when applicable, from legally authorized representatives.

### 2.3. Questionnaire Design

The questionnaire was specifically developed to capture culturally relevant dietary habits related to wheat consumption in Pakistan. It included structured questions on meal frequency, types of wheat-based products consumed, flour preferences, chapati consumption pattern, and general dietary habits related to brain health.

The questionnaire was designed following established principles for structured consumer research instruments as described by Macfie & Thomson [15], with emphasis on clarity, consistency, and cultural appropriateness. Interviewer-guided administration was used to ensure uniform interpretation of questions and to minimize variability in data collection.

### 2.4. Statistical Analysis

All questionnaire responses were anonymized and entered into a secure database. Data analysis was performed using GraphPad Prism (v. 4.00 GraphPad Software, Inc., San Diego, CA, USA). Descriptive statistics were used to summarize the characteristics of the study population. Categorical variables were expressed as frequencies and percentages. However, continuous variables, such as age and body mass index (BMI) are described as mean ± standard deviation (SD).

To explore potential differences between HLT and PwD, contingency table analyses were performed using the Pearson’s chi-square ($\chi^{2}$) test or Fisher’s exact test where appropriate. Due to the exploratory nature of the study and the relatively small sample size (*n* = 30 per group), the statistical analysis was primarily intended to identify trends rather than establish causal relationships. A *p*-value < 0.05 was considered statistically significant.

## 3. Results

### 3.1. Demographics and Health Overview of Pakistani Population

As summarized in Table 1, a total of 144 Pakistani participants were included in the study. The sample consisted of 23.6% males and 76.4% females. The mean age was 38.7 ± 14.7 years, with most individuals between 31 and 40 years (36.8%), followed by 21–30 years (34.0%), while 25.0% were older than 50 years and 4.2% between 41 and 50 years.

Regarding marital status, 71.5% of participants were married, whereas 28.5% were unmarried. Most respondents belonged to the higher income group (>50 k; 63.2%), while 36.8% reported a monthly income below 50 k. Educational attainment varied: 39.6% held a master’s degree, 29.9% a bachelor’s degree, 24.3% had a PhD, 4.9% a high school qualification, while only 1.4% reported no formal education.

Allergies were reported by 9.7% of the population. The mean BMI at enrolment was 24.6 ± 4.7 kg/m^2^, with 49.3% classified as normal weight, 30.6% overweight, 12.5% obese, and 7.6% underweight.

Daily physical activity patterns showed that 34.7% of participants engaged in cooking or household tasks, 24.3% performed brisk walking, and 15.3% attended a gym, while 19.4% reported no regular physical activity. Cleaning or gardening was practiced by 6.3% of participants.

With respect to medical conditions, 62.5% of individuals reported no diagnosed pathology. Among the remaining participants, the most prevalent conditions included hypertension (13.9%), obesity (13.9%), diabetes mellitus (9.7%), and irritable bowel disease (6.3%). Less frequent conditions were cardiovascular disease (3.5%), gluten intolerance (2.1%), dementia (2.1%), celiac disease (0.7%), and other unspecified disorders (12.5%). Regarding disease duration, 23% of the population had been diagnosed for less than 10 years, whereas 9% had been living with their condition for more than 10 years. When asked about potential causes of their illnesses, 36% attributed their condition to poor diet, 9% to aging, and 6% specifically to the consumption of refined flour.

Symptoms affecting daily routine were uncommon, with 68.1% reporting no symptoms. The most frequently reported issues were memory problems related to recent events (11.1%), reduced concentration (6.3%), and increasing confusion (4.2%). Other symptoms, such as personality changes, apathy, loss of everyday functioning, or planning difficulties, were each reported by a few participants.

Self-perceived brain health was rated as good or excellent by 66.6% of participants, while 28.5% rated it as average. Only 4.9% of individuals assessed their brain health as poor or bad. Use of multivitamins was reported by 47.9% of participants.

### 3.2. Wheat Product Intake, Flour Preferences, and Chapati Consumption Habits in Pakistan

**Figure 1:**
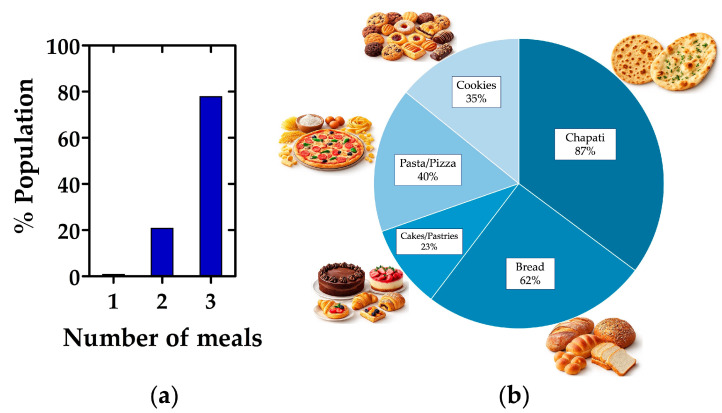
Dietary survey results from a Pakistani population. (**a**) Distribution of respondents by the number of daily meals; (**b**) frequency of consumption of common wheat-based food products, including chapati, bread, pasta/pizza, cakes/pastries, and cookies.

The majority of participants reported consuming three meals per day (78%), while 21% indicated eating twice daily and only 1% reported eating once a day (Figure 1a). This pattern is in line with what might be expected in a “standard” three-meals-a-day dietary scheme. However, in the context of dietary studies in Pakistan, evidence suggests considerable variability in meal frequency and diet composition depending on socioeconomic status, urban vs. rural residence, and seasonal availability of food [16,17]. The predominance of a three-meals-per day pattern may reflect relative food security and stable daily routines in the surveyed population. Nonetheless, meal frequency alone does not provide information about portion sizes, energy density, or nutritional balance. Given the high reliance on wheat-based foods, it is plausible that energy intake is sufficient, but nutrient density (especially in terms of fiber, micronutrients, and protein quality) remains suboptimal.

Among Pakistani respondents, the data show a high reliance on wheat-based foods in their daily diet. Chapati was the most frequently consumed item (87%), followed by bread (62%). Other commonly consumed wheat products included pasta or pizza (40%), cookies (35%), and cakes or pastries (23%) (Figure 1b). These findings highlight the central role of wheat products in the dietary patterns of the Pakistani population surveyed. The predominance of wheat-based staples aligns with findings from recent community-based research among Pakistani adults, which identified distinct dietary patterns, including “discretionary” or “processed food” patterns, alongside more traditional ones [18]. National-level data confirm that wheat remains the main staple crop and food source in Pakistan, contributing substantially to the average household caloric intake [19,20]. Moreover, the non-negligible proportion of participants consuming processed wheat-based foods (pasta/pizza, cookies, pastries) suggests a “hybrid diet” combining traditional staples (chapati, bread) with modern or convenience foods. This pattern aligns with recent observations of nutrition transition in urban and peri-urban Pakistani populations, characterized by increased consumption of processed and discretionary foods [18,21].

**Figure 2:**
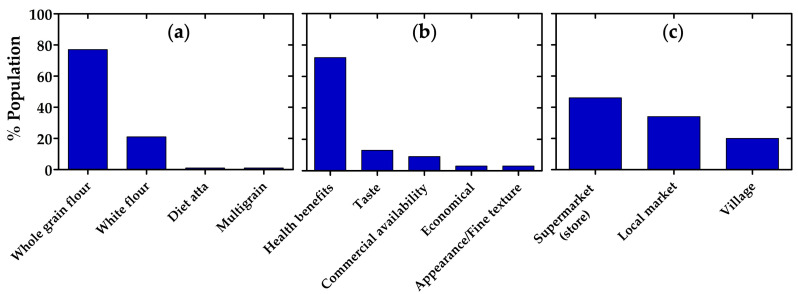
Survey results from a Pakistani population regarding flour preferences and purchasing habits. (**a**) Types of flour commonly consumed; (**b**) primary factors influencing flour selection; (**c**) main locations where flour is purchased.

Most respondents reported using whole grain wheat flour (77%), making it predominant choice, whereas white flour accounted for 21% of use, and “diet atta” and multigrain flour were each selected by 1% of participants (Figure 2a). The preference for whole wheat flour within the Pakistani population was largely motivated by health considerations, with 72% indicating that perceived health benefits were the primary reason for their choice, despite most respondents reporting limited awareness of the nutraceutical components of whole grain flour, the survey indicated a general preference for whole wheat flour; notably, many participants stated they were already using whole wheat flour, and a substantial proportion expressed willingness to switch to it if advised by healthcare professionals. Other factors played a comparatively minor role, including taste (13%), commercial availability (9%), economical price (3%), and appearance or fine texture (3%) (Figure 2b). Regarding purchasing patterns, 46% of respondents typically obtained wheat flour from supermarkets, while 34% relied on local markets and 20% sourced it from village-level vendors. Together, these findings illustrate not only a clear inclination toward whole grain flour but also the strong influence of health-related motivations on consumer decisions (Figure 2c).

**Figure 3:**
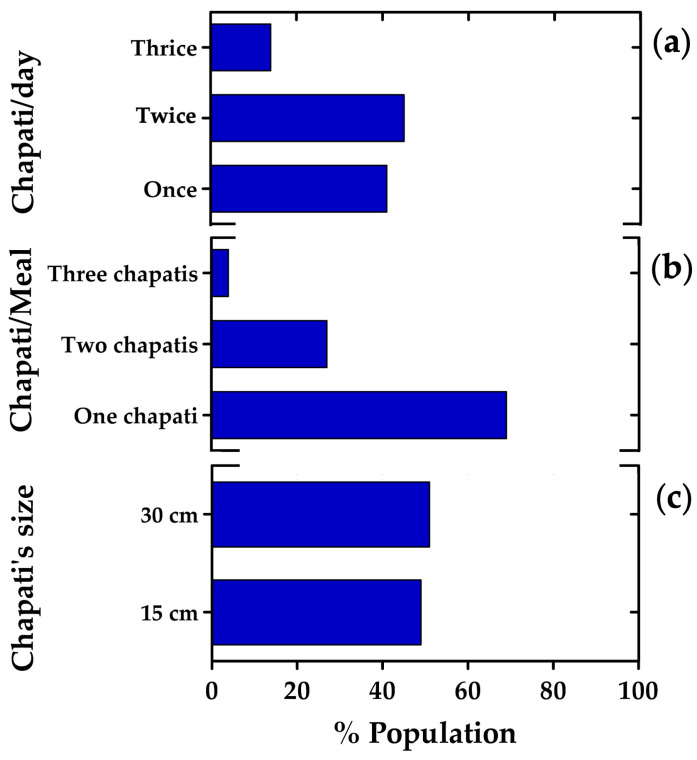
Chapati consumption patterns in a Pakistani population. (**a**) Frequency of daily chapati intake (in the insert, chapati prepared from whole wheat flour); (**b**) number of chapatis typically consumed per meal; (**c**) preferred chapati size.

Given the high consumption of chapati within the Pakistani population (see Figure 1b), additional analyses were conducted to explore its role in daily dietary habits. The majority of respondents reported eating chapati once (41%) or twice (45%) per day, while a smaller proportion consumed it three times daily (14%) (Figure 3a). In terms of portion size, most individuals typically ate the equivalent of one chapati per meal (69%), whereas 27% consumed two and only 4% consumed three (Figure 3b). Preferences regarding chapati dimensions were almost evenly split: 49% favored a 15 cm chapati, while 51% preferred the larger 30 cm size (Figure 3c). These findings highlight not only the widespread integration of chapati into daily eating patterns but also the consistency in portion size and dimensional preferences among consumers.

### 3.3. Demographic and Clinical Profile of HLT vs. PwD Enrolled in the Study

The demographic and clinical characteristics of HLT and PwD are presented in Table 2 and compared using Pearson’s chi-square tests, with Fisher’s exact tests applied when appropriate. No significant differences were observed between groups for gender distribution, age group, income level, or multivitamin use (all *p* > 0.05). The gender distribution was comparable between groups, with a slightly higher proportion of females in both cohorts (HLT: 63.3%; PwD: 60.0%). PwD were generally older, with 60.0% being older than 50 years compared with 50.0% in the HLT group.

All participants were married. A higher percentage of PwD belonged to the lower–income group (<50k), representing 20.0% of the cohort compared with 10.0% among HLT. Educational attainment differed markedly between groups: 96.7% of HLT reported having formal education, whereas only 60.0% of PwD did so ($\chi^{2}=$ 10.24, *p* = 0.001).

Daily physical activity was substantially reduced in the dementia group. While 96.7% of HLT engaged in regular physical activity, only 36.7% of PwD reported doing so ($\chi^{2}=$ 21.70, *p* < 0.0001). Consistent with this, 63.3% of PwD reported being physically inactive compared with only 3.3% of HLT.

Regarding comorbidities, hypertension was significantly more prevalent among PwD (53.3%) compared with HLT (16.7%) ($\chi^{2}=$ 8.57, *p* = 0.003). PwD also reported a higher prevalence of other medical conditions (26.7% vs. 6.7%; *p* < 0.05), whereas the absence of comorbidities was significantly more frequent among HLT (60.0% vs. 16.7%; $\chi^{2}=$ 11.78, *p* < 0.001). No significant differences were observed for cardiovascular disease, diabetes mellitus, or obesity (all *p* > 0.05).

With regard to the subjective assessment of disease severity, the questionnaire included seven items ranging from “no cognitive decline” to “very severe cognitive decline.” Although all patients had mild-to-moderate dementia, their self-perceived ratings were highly variable. This altered self-perception reflects another well-known feature of the disease, namely impaired awareness of illness.

The average age at clinical diagnosis in the dementia group was 58.3 ± 15.4 years, with the highest proportion diagnosed between 51 and 60 years (36.0%).

### 3.4. Eating Habits in Life (Lifelong Diet)

During the survey, both the HLT and individuals with dementia were asked to describe the type of diet they had followed throughout their lives, prior to the onset of dementia. Participants with dementia reported having historically consumed a more monotonous diet, largely based on refined flours, whereas HLT tended to report a higher intake of whole grain products and more varied meals.

**Figure 4:**
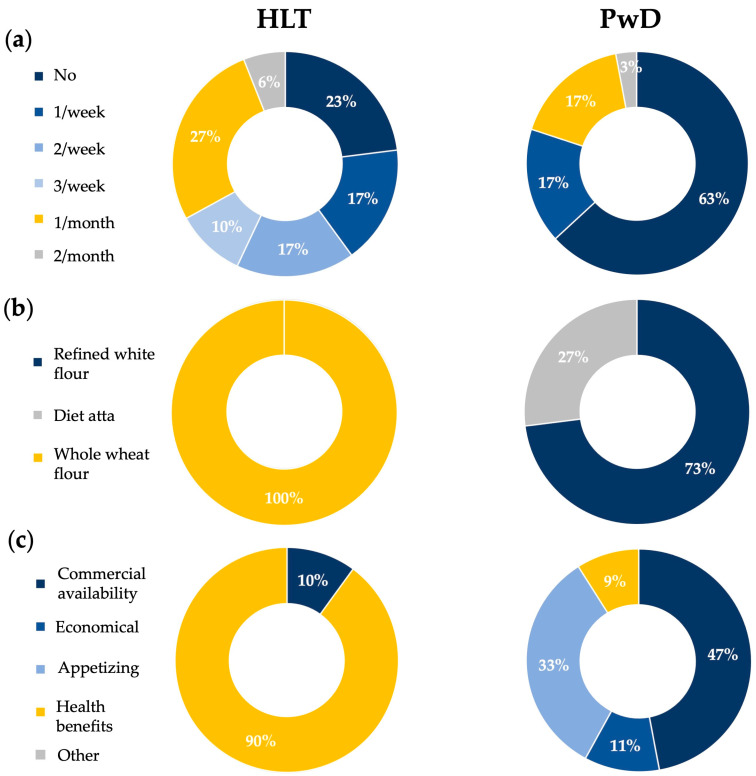
Comparison of food consumption habits and flour preferences between HLT and PwD. (**a**) Frequency of outdoor dining; (**b**) type of flour typically used; (**c**) main factors influencing flour choice. HLT: healthy participants; PwD: patients with dementia.

Figure 4a shows the frequency of outdoor dining. As can be seen, clear differences emerge between HLT and PwD. HLT show a more varied distribution, with a substantial proportion reporting monthly (27%) or no outdoor meals (23%), and over one third indicating weekly outdoor meals (1–3 times/week: 44%). In contrast, individuals with dementia predominantly report no outdoor meal consumption (63%), with only a minority indicating occasional participation, either once per week or once per month (17% each). Notably, none of the PwD report consuming outdoor meals more than once per week, whereas this pattern is relatively common among HLT (27%). The frequency of outdoor meals across the lifespan differed significantly between HLT and PwD ($\chi^{2}$(5) = 14.56, *p* = 0.012). Because of small expected counts, Fisher’s exact test was also performed and confirmed the significance of the association (*p* = 0.006). These findings align with the existing literature suggesting that engagement in social and outdoor activities tends to decline in dementia, potentially reflecting both reduced functional autonomy and diminished participation in lifestyle habits established earlier in life [22,23].

Both HLT and PwD group subjects were asked which type of flour they preferred for preparing chapati at home and for how long they had been consuming it. Participants reported using the same type of flour since the beginning of their habitual diet. As shown in Figure 4b, all HLT reported the use of whole wheat flour, whereas PwD predominantly reported long-term consumption of refined white flour (73%) or “diet atta” (27%). Pearson’s chi-square test revealed a highly significant difference between groups ($\chi^{2}$(2) = 60.0, *p* < 0.0001), and Fisher’s exact test was also performed and confirmed the statistical significance of the association (*p* < 0.0001). These findings are consistent with the scientific literature indicating that diets richer in whole grains are more common among healthier populations, while more refined and less varied dietary pattern are frequently observed among individuals with cognitive decline [6,7,8,24].

Furthermore, both the HLT group and individuals with dementia were asked why they preferred a specific type of flour for preparing chapati. As shown in Figure 4c, HLT predominantly chose whole wheat flour due to its perceived health benefits (90%), including higher fiber content and the presence of nutraceuticals and vitamins. In contrast, PwD tended to prefer refined white flour, for greater commercial availability (47%), more appealing color and appearance (33%), and lower cost (11%). Pearson’s chi-square test showed a significant association between participant group and reported motivations ($\chi^{2}$(3) = 37.86, *p* < 0.0001), confirmed by Fisher’s exact test (*p* < 0.0001). These patterns align with the scientific literature indicating that healthier populations are more likely to prioritize nutritional value, whereas individuals with cognitive decline may rely more on convenience, sensory appeal, and affordability when making dietary choices [25,26,27,28]. However, the 9% of dementia patients who reported choosing their flour for its beneficial effects are those who use “diet atta”, a blended flour formulated for low-calorie diets. Such mixes are typically lower in carbohydrates and richer in fiber, protein, and other nutrients, which may explain why this small subset of patients associated their flour choice with health benefits.

### 3.5. Dietary Patterns Associated with Brain Health

**Figure 5:**
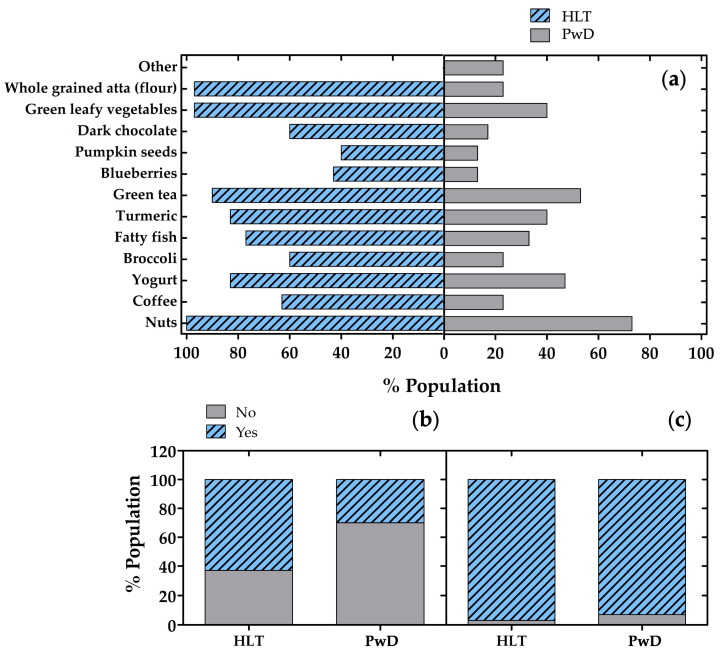
Dietary habits and consumption of neuroprotective foods among HLT and PwD. (**a**) Percentage of participants reporting regular consumption of selected brain-healthy foods and ingredients; (**b**) differences between HLT and PwD in the consumption of additional foods specifically chosen to support brain function; (**c**) awareness of the benefits of nutraceuticals contained in whole grains. HLT: healthy participants; PwD: patients with dementia.

The consumption of foods commonly associated with brain health was compared between HLT and PwD using Pearson’s chi-square tests and Fisher’s exact tests. Figure 5a shows clear differences between HLT and PwD in their daily consumption of foods typically associated with better cognitive health. Overall, HLT reported a higher daily consumption of coffee, broccoli, fatty fish, turmeric, blueberries, pumpkin seeds, dark chocolate, green leafy vegetables, and whole-grained atta compared with PwD (all *p* < 0.05). The strongest association was observed for whole-grained atta consumption ($\chi^{2}=$ 26.07, *p* < 0.0001). Conversely, the lower intake of these foods among dementia patients may reflect long-term dietary patterns that contribute to cognitive decline, or alternatively, reduced ability to maintain healthy eating habits after the onset of symptoms. PwD more frequently selected the category “other” foods (*p* ≈ 0.01). In contrast, the consistently higher consumption reported by HLT reinforces the growing evidence that sustained adherence to nutrient-dense dietary patterns are associated with better brain aging and reduced dementia risk. No statistically significant differences between groups were observed for nuts, yogurt, or green tea consumption (*p* > 0.05).

A substantial difference between HLT and PwD in their use of additional foods, specifically chosen to support healthy brain function, is shown in Figure 5b. While 63% of HLT report consuming other brain-supportive foods, only 30% of PwD do so, suggesting that HLT participants are more attentive to incorporating targeted dietary elements into their daily routines. Pearson’s chi-square test indicated a significant association between group and response ($\chi^{2}$(1) = 6.67, *p* = 0.0098). Fisher’s exact test confirmed the significance of this difference (*p* = 0.018). Notably, among the HLT respondents who answered positively, the most commonly mentioned items were olive oil and honey, both frequently highlighted in the scientific literature for their antioxidant, anti-inflammatory, and neuroprotective properties [29,30,31,32]. In contrast, the higher percentage of negative responses among dementia patients may reflect reduced dietary variety, diminished nutritional awareness, or changes in eating habits that often accompany cognitive decline.

Figure 5c shows that awareness of the benefits of nutraceuticals in whole grains is high in both groups, though slightly higher among HLT (97%) compared to PwD (93%). This small difference may indicate that HLT are marginally more exposed to or engaged with nutritional information, while PwD may experience reduced access to such knowledge or memory-related difficulties in recalling it. Therefore, no statistically significant difference was observed between groups. Pearson’s chi-square test showed no significant association between participant group and awareness ($\chi^{2}$(1) = 0.35, *p* = 0.55). Given the presence of small expected counts, Fisher’s exact test was also performed and confirmed the lack of a significant difference (*p* = 1.00). According to the recent scientific literature, whole grains contain a variety of nutraceutical compounds, including dietary fiber, vitamins, minerals, phenolic acids, lignans, and antioxidants, that contribute to reduced inflammation, improved metabolic health, and potentially enhanced cognitive function [33,34,35,36,37,38]. These components support vascular health and help counteract oxidative stress, both of which play essential roles in maintaining brain integrity [37,39,40]. The high level of awareness observed in both groups suggests that the perceived health value of whole grains is broadly recognized, even though actual dietary habits may differ.

Importantly, the results should not be interpreted as suggesting that whole wheat flour intake alone determines cognitive health outcomes. Participants in the HLT group also reported higher consumption of other foods commonly associated with neuroprotective dietary patterns, including nuts, green leafy vegetables, and antioxidant-rich foods. This suggests that whole wheat flour intake may represent one component of a broader healthy dietary profile rather than an isolated protective factor. This observation is consistent with the current literature on dietary patterns such as the Mediterranean and MIND diets, where multiple dietary components interact synergistically to support brain health [41,42,43,44,45,46,47,48].

### 3.6. Attribution of the Causes of Dementia

**Figure 6:**
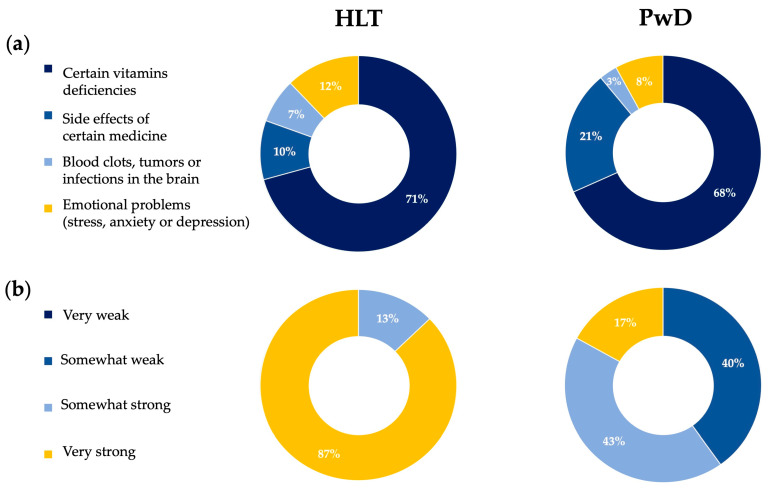
(**a**) Percentage of respondents identifying potential causes of memory problems; (**b**) differences between HLT participants and PwD in their sense of belonging to the local community. HLT: healthy participants; PwD: patients with dementia.

Figure 6a shows that a large majority of HLT participants (71%) and a similarly high proportion of people with dementia (68%) believe that “certain vitamin deficiencies” are a primary cause of dementia, far more than other potential causes such as side effects of medications, brain infections/tumors, or emotional problems. No statistically significant differences were observed between groups for any of the proposed causes. Pearson’s chi-square tests yielded non-significant results for all comparisons (all *p* > 0.05), and Fisher’s exact tests confirmed the absence of significant associations between group and perceived causes of dementia. This indicates that both HLT and PwD often attribute dementia to modifiable nutritional factors. Such intuitions are not unfounded: growing scientific evidence supports a link between low vitamin status and cognitive decline. For instance, a 2023 meta-analysis found that deficiency of vitamin D is associated with a ~1.42-fold increased risk of dementia and a ~1.57-fold increased risk of Alzheimer’s disease [49]. Similarly, a 2022 meta-analysis of 95 studies with over 46 000 participants showed that supplementation with B vitamins (especially folate) may slow cognitive decline and reduce the risk of incident dementia among older adults without dementia [50]. Moreover, clinical research has demonstrated that treating vitamin B12 deficiency in patients with mild cognitive impairment can lead to measurable improvements in cognition [51,52]. In addition, neuroimaging data indicate that B12 deficiency is associated with brain atrophy (e.g., hippocampal shrinkage), a structural correlate of dementia [53]. Therefore, the high frequency with which participants cite vitamin deficiencies as a cause of dementia appears broadly consistent with current scientific understanding of modifiable risk factors, though not all vitamin deficiencies lead to dementia, and supplementing vitamins does not guarantee prevention.

### 3.7. Sense of Community and Social Belonging

Figure 6b shows a stark difference between HLT participants and PwD in their reported sense of belonging to the local community: 87% of HLT describe their sense of belonging as “*very strong*”, whereas only 17% of PwD do so, with 43% of patients rating it “*somewhat strong*” and 40% “*somewhat weak*”. Pearson’s chi-square test revealed a highly significant association between participant group and perceived sense of community belonging ($\chi^{2}$(2) = 27.13, *p* < 0.0001) Because of small expected counts, Fisher’s exact test was also performed and confirmed the statistical significance of the association (*p* < 0.0001). This suggests that people living with dementia often experience a significantly diminished feeling of social belonging compared to cognitively HLT, a finding that may reflect the well-documented risk of social isolation, diminished social participation, and reduced opportunities for engagement experienced by this population. This pattern resonates with the recent scientific literature. For example, a 2023 study reported that participation in community-based social initiatives like *Dementia Cafés* gave people with dementia and their caregivers an enhanced sense of belonging and purpose, an important boost to their social health and well-being [54]. Similarly, a 2024 ecological momentary assessment of social interaction in long-term care showed that many people with dementia spend large portions of the day without any social contact: in the sample, no interaction occurred during ~72% of observation periods [55]. Moreover, a recent meta-analysis demonstrated that feelings of loneliness and social isolation are highly prevalent among individuals with mild cognitive impairment or dementia, pointing to social disconnectedness as a common, serious problem [56]. These findings help explain why so few patients in our sample report a “*very strong*” sense of community: their disease often disrupts social networks, reduces opportunities for meaningful interaction, and undermines feelings of inclusion and belonging. At the same time, the positive impact of community-based support structures (like *Dementia Cafés*, peer–support groups, inclusive neighborhood efforts) highlights that strengthening social belonging is not only desirable but potentially meaningful for well-being and care. Indeed, our study revealed that 80% of patients either never dined out or did so only once, while 83% reported a weak or very weak sense of community and social belonging, suggesting a strong association between eating out and feelings of loneliness (Figure 4a and Figure 6b).

## 4. Discussion

Although the health benefits of whole grain consumption have been widely reported [11], limited data are available on their potential association with cognitive health within specific cultural dietary contexts. In Pakistan, chapati is a staple food, and the type of flour used for its preparation represents an important determinant of whole grain intake.

This cross-sectional study provides insight into the potential role of dietary whole wheat flour intake in Pakistan as a modifiable factor relevant to long-term dementia prevention. Although causality cannot be inferred, the findings should be interpreted within the growing body of evidence linking whole grains, dietary fiber, and bioactive compounds to mechanisms involved in cognitive aging and neurodegeneration. Furthermore, the results should therefore be interpreted within the context of broader dietary patterns. Participants reporting higher whole wheat flour consumption also tended to report grater intake of other foods associated with brain-healthy diets, suggesting that whole wheat flour intake may represent one component of a generally healthier dietary profile rather than an isolated determinant of cognitive health.

Whole wheat flour is a rich source of dietary fiber, B-group vitamins, minerals, and phenolic compounds, which are largely removed during refining. Previous nutritional analyses have demonstrated that whole grains contain antioxidant and anti-inflammatory components capable of modulating oxidative stress and systemic inflammation, both of which are central to dementia pathophysiology [57,58]. These mechanisms are particularly relevant given the established role of chronic low-grade inflammation and oxidative damage in neuronal dysfunction and synaptic loss [59].

Furthermore, dietary fiber from whole grains contributes to improved metabolic regulation, including glycemic control and lipid profiles, which are known risk modifiers for vascular dementia and Alzheimer’s disease [34,60]. The association between metabolic disorders and cognitive decline has been consistently reported, supporting the biological plausibility of dietary interventions targeting staple foods [10].

Recent evidence suggests that whole grain intake may influence brain health through modulation of the gut–brain axis. Whole wheat-derived fibers and polyphenols can alter gut microbiota composition, leading to increase production of short-chain fatty acids with neuroprotective and anti-inflammatory properties [11,61]. Dysbiosis and intestinal permeability have been increasingly implicated in neurodegenerative processes, reinforcing the relevance of dietary patterns rich in unrefined plant foods.

In addition, experimental and toxicological studies indicate that whole grain phytochemicals may mitigate neurotoxicity and hormonal dysregulation linked to cognitive impairment [62,63]. These findings support the hypothesis that long-term consumption of whole wheat flour could contribute to neuroprotection through multiple converging biological pathways.

While randomized controlled trials directly assessing whole wheat flour and dementia outcomes remain limited, observational and interventional evidence supports the role of plant-based, fiber-rich diets in reducing cognitive decline. Reviews and systematic analyses indicate that diets emphasizing whole grains are associated with better cognitive performance and reduced dementia incidence, although heterogeneity and methodological limitations persist [7,64,65]. Recent clinical and pharmacological perspectives also highlight nutrition as a key component of multidomain dementia prevention strategies [6,66].

In Pakistan, wheat flour constitutes a primary caloric source, making the distinction between refined and whole wheat flour particularly important at a population level. The widespread consumption of refined wheat may contribute to micronutrient insufficiencies and metabolic risk, whereas greater reliance on whole wheat flour could offer a feasible, culturally acceptable intervention. Food science research supports the nutritional superiority of whole wheat products and emphasizes the need for improved processing and consumer awareness to enhance acceptability and health impact [67,68]. Therefore, understanding the role of staple foods in long-term dietary exposures may be particularly important when designing culturally relevant strategies for dementia prevention.

## 5. Conclusions

In conclusion, this study adds to the emerging evidence that whole wheat flour intake may represent one potentially relevant component of broader dietary patterns associated with long-term brain health. Given the central role of wheat in the Pakistani diet, promoting whole wheat flour consumption represents a potentially impactful and scalable strategy within broader dementia prevention frameworks.

Several limitations should be considered when interpreting the results. Due to the cross-sectional design, current dietary habits may not fully reflect long-term dietary patterns associated with dementia development. In addition, although caregivers or family members assisted patients when necessary, recall bias cannot be completely excluded in participants with cognitive impairment. The sample size was determined based on feasibility and the exploratory nature of the study rather than a formal a priori power calculation. Recruitment was limited to a defined clinical setting and time period. Consequently, the study should be considered a pilot investigation intended to identify potential dietary patterns that may warrant further investigation in larger prospective studies.

Future research should prioritize prospective cohort studies and randomized dietary interventions in South Asian populations, incorporating standardized cognitive assessments and biomarkers of inflammation, oxidative stress, and gut microbiota composition, to better elucidate the potential contribution of long-term replacement of refined wheat with whole wheat flour to dementia prevention. As participants in the present study were recruited from a private hospital setting and were generally well educated and health-aware, inclusion of socioeconomically diverse populations will be essential to enhance the generalizability of these findings.

Despite these limitations, the present findings provide preliminary evidence suggesting that dietary patterns involving whole wheat consumption may be associated with cognitive health. Further large-scale longitudinal studies are needed to clarify whether such dietary habits play a direct role in dementia prevention.

## Figures and Tables

**Table 1:** Demographic profile and health conditions of the subjects (*n* = 144) examined.

Clinical Features:	*n* (%)	Clinical Features:	*n* (%)
*Gender*		*Pathologies*	
Males	34 (23.6)	Hypertension	20 (13.9)
Females	110 (76.4)	Cardiovascular disease	5 (3.5)
*Age (years)*	38.7 ± 14.7	Diabetes mellitus	14 (9.7)
21–30	49 (34.0)	Irritable bowel disease	9 (6.3)
31–40	53 (36.8)	Obesity	20 (13.9)
41–50	6 (4.2)	Celiac disease	1 (0.7)
>50	36 (25.0)	Gluten intolerance	3 (2.1)
*Marital status*		Dementia	3 (2.1)
Unmarried	41 (28.5)	Other	18 (12.5)
Married	103 (71.5)	None	90 (62.5)
*Income group*		*Symptoms experienced in daily routine*	
<50k	53 (36.8)	Language problems	0 (0.0)
>50k	91 (63.2)	Increased confusion	6 (4.2)
*Education*		Reduced concentration	9 (6.3)
None	2 (1.4)	Personality or behavior issues	2 (1.4)
High school	7 (4.9)	Loss of ability to do everyday tasks	6 (4.2)
Bachelor’s degree	43 (29.9)	Apathy and withdrawal or depression	2 (1.4)
Master’s degree	57 (39.6)	Not recognizing friends and family	0 (0.0)
PhD	35 (24.3)	Problems with planning and decision-making	4 (2.8)
*Allergies*		Not remembering where they live or where they are	1 (0.7)
Yes	14 (9.7)	Memory problems, particularly remembering recent events	16 (11.1)
No	130 (90.3)	Other	0 (0.0)
*BMI at enrolment* (kg/m^2^)	24.6 ± 4.7	None	98 (68.1)
Underweight	11 (7.6)	*Brain health*	
Normal	71 (49.3)	Bad	2 (1.4)
Overweight	44 (30.6)	Poor	5 (3.5)
Obese	18 (12.5)	Average	41 (28.5)
*Daily physical activity*		Good	67 (46.5)
Gym	22 (15.3)	Excellent	29 (20.1)
Brisk walk	35 (24.3)		
Cleaning or gardening	9 (6.3)	**Use of multivitamins:**	
Cooking and washing up	50 (34.7)	Yes	69 (47.9)
None	28 (19.4)	No	75 (52.1)

BMI: body mass index.

**Table 2:** Demographic profile and health conditions of PwD (*n* = 30) vs. HLT (*n* = 30).

Clinical Features:	HLT *n* (%)	PwD *n* (%)	$\chi^{2}$	*p* (Pearson’s Chi-Square)	*p* (Fisher’s Exact Test)
*Gender*			0.07	0.79	1.00
Males	11 (36.7)	12 (40.0)			
Females	19 (63.3)	18 (60.0)			
*Age*			0.67	0.41	0.60
<50	15 (50.0)	12 (40.0)			
>50	15 (50.0)	18 (60.0)			
*Marital status*					
Unmarried	0 (0.0)	0 (0.0)			
Married	30 (100.0)	30 (100.0)			
*Income group*			1.18	0.28	0.47
<50k	3 (10.0)	6 (20.0)			
>50k	27 (90.0)	24 (80.0)			
*Education*			10.24	0.001	0.002
Yes	29 (96.7)	18 (60.0)			
No	1 (3.3)	12 (40.0)			
*Daily physical activity*			21.70	<0.0001	<0.0001
Yes	29 (96.7)	11 (36.7)			
No	1 (3.3)	19 (63.3)			
*Other pathologies*					
Hypertension	5 (16.7)	16 (53.3)	8.57	0.003	0.006
Cardiovascular disease	0 (0.0)	2 (6.7)	2.07	0.15	0.49
Diabetes mellitus	7 (23.3)	9 (30.0)	0.34	0.56	0.79
Irritable bowel disease	0 (0.0)	0 (0.0)			
Obesity	3 (10.0)	0 (0.0)	3.16	0.07	0.24
Celiac disease	0 (0.0)	0 (0.0)			
Gluten intolerance	0 (0.0)	0 (0.0)			
Other	2 (6.7)	8 (26.7)	4.32	0.038	0.046
None	18 (60.0)	5 (16.7)	11.78	0.0006	0.001
*Symptoms experienced in daily routine*					
Language problems	0 (0.0)	19 (63.3)			
Increased confusion	0 (0.0)	16 (53.3)			
Reduced concentration	0 (0.0)	1 (3.3)			
Personality or behavior issues	0 (0.0)	8 (26.7)			
Loss of ability to do everyday tasks	0 (0.0)	8 (26.7)			
Apathy and withdrawal or depression	0 (0.0)	1 (3.3)			
Not recognizing friends and family	0 (0.0)	5 (16.7)			
Problems with planning and decision-making	0 (0.0)	12 (40.0)			
Not remembering where they live or where they are	0 (0.0)	5 (16.7)			
Memory problems, particularly remembering recent events	0 (0.0)	2 (6.7)			
Other	0 (0.0)	0 (0.0)			
None	30 (100.0)	0 (0.0)			
*Stage of dementia*					
No cognitive decline	30 (100.0)	0 (0.0)			
Very mild cognitive decline	0 (0.0)	3 (10.0)			
Mild cognitive decline	0 (0.0)	8 (26.7)			
Moderate cognitive decline	0 (0.0)	7 (23.3)			
Moderately severe cognitive decline	0 (0.0)	7 (23.3)			
Severe cognitive decline	0 (0.0)	5 (16.7)			
Very severe cognitive decline	0 (0.0)	0 (0.0)			
*Average age of clinical diagnosis*		58.3 ± 15.4			
<30		2 (6.7)			
31–40		2 (6.7)			
41–50		5 (16.7)			
51–60		11 (36.0)			
61–70		4 (13.3)			
>70		6 (20.0)			
*Use of multivitamins*			1.07	0.30	0.44
Yes	16 (53.3)	12 (40.0)			
No	14 (46.7)	18 (60.0)			

HLT: healthy participants; PwD: patients with dementia.

## Data Availability

The raw data supporting the conclusions of this article will be made available by the authors on request.
